# Functions of Real-Life Conversational Time Travel in the Context of Healthy Aging

**DOI:** 10.1093/geroni/igaa057.2120

**Published:** 2020-12-16

**Authors:** Burcu Demiray, Minxia Luo, Mike Martin

**Affiliations:** University of Zurich, Zurich, Zurich, Switzerland

## Abstract

Using smartphone sensing in real life, we examined conversational time travel (i.e., talking about the personal past versus future), its functions and relation with positive affect (i.e., laughing behavior). We used the Electronically Activated Recorder (audio recorder that periodically records snippets of ambient sounds and speech) and collected a random sample of over 30,000 sound snippets (30 seconds long) from 61 young and 48 healthy older adults across four days. We transcribed and manually coded participants’ speech. Multilevel models conducted in R showed that individuals tended to talk about their past with more social functions (e.g., give advice), whereas talked about their future for more directive purposes (e.g., planning). Age group differences were minimal. We also found that individuals laughed two times more while talking about their past than their future. Results are discussed in relation to the functions of mental/conversational time travel in the context of healthy aging.

